# Vehicle Logo Recognition Based on Enhanced Matching for Small Objects, Constrained Region and SSFPD Network

**DOI:** 10.3390/s19204528

**Published:** 2019-10-18

**Authors:** Ruikang Liu, Qing Han, Weidong Min, Linghua Zhou, Jianqiang Xu

**Affiliations:** 1 School of Information Engineering, Nanchang University, Nanchang 330031, China; liuruikanglin@163.com (R.L.); hanqing@ncu.edu.cn (Q.H.); z199513z@163.com (L.Z.); xjq@ncu.edu.cn (J.X.); 2 School of Software, Nanchang University, Nanchang 330047, China; 3 Jiangxi Key Laboratory of Smart City, Nanchang 330047, China

**Keywords:** vehicle logo recognition, enhanced matching, constrained region, SSFPD, robotic systems

## Abstract

Vehicle Logo Recognition (VLR) is an important part of vehicle behavior analysis and can provide supplementary information for vehicle identification, which is an essential research topic in robotic systems. However, the inaccurate extraction of vehicle logo candidate regions will affect the accuracy of logo recognition. Additionally, the existing methods have low recognition rate for most small vehicle logos and poor performance under complicated environments. A VLR method based on enhanced matching, constrained region extraction and SSFPD network is proposed in this paper to solve the aforementioned problems. A constrained region extraction method based on segmentation of the car head and car tail is proposed to accurately extract the candidate region of logo. An enhanced matching method is proposed to improve the detection performance of small objects, which augment each of training images by copy-pasting small objects many times in the unconstrained region. A single deep neural network based on a reduced ResNeXt model and Feature Pyramid Networks is proposed in this paper, which is named as Single Shot Feature Pyramid Detector (SSFPD). The SSFPD uses the reduced ResNeXt to improve classification performance of the network and retain more detailed information for small-sized vehicle logo detection. Additionally, it uses the Feature Pyramid Networks module to bring in more semantic context information to build several high-level semantic feature maps, which effectively improves recognition performance. Extensive evaluations have been made on self-collected and public vehicle logo datasets. The proposed method achieved 93.79% accuracy on the Common Vehicle Logos Dataset and 99.52% accuracy on another public dataset, respectively, outperforming the existing methods.

## 1. Introduction

Vehicle Logo Recognition (VLR) is an important part of vehicle behavior analysis and can provide supplementary information for vehicle identification, which is an essential research topic in robotic systems. In recent years, the increasing popularity of traffic monitoring information systems has resulted in the rapid development of vision-based vehicle identification technology, which has become a research hotspot in the field of robotic systems. Vehicle identification plays an important role in maintaining traffic and public security management. Traffic accidents and car-related crimes often occur in the real world. Usually, a contaminated or obscured vehicle license plate will cause an unstable performance of the license plate recognition technology. In addition, some individuals and criminals even make fake plate vehicles to evade taxes and disrupt the normal transportation order. Therefore, obtaining vehicle information merely through license plate cannot constantly address the actual needs. Accordingly, vehicle logo, which is a key feature of vehicles, can assist in vehicle identification. Similar to license plate recognition, vehicle logo recognition (VLR) is also one of the important technologies to achieve intelligent transportation systems.

VLR can provide complementary information for vehicle identification [1,2] in robotic systems. To effectively combat car-related crimes, an increasing number of researchers have devoted effort to design efficient vehicle logo recognition methods. The most popular methods include two steps: vehicle logo candidate region extraction and logo recognition. However, the existing VLR methods continue to have difficulty in detecting small logos and recognizing logos under complex environmental situations. Thus, recognizing the small objects is a fundamental challenge in computer vision. Many state-of-the-art detectors typically perform well in large object-detection tasks but often have low detection accuracy for small traffic objects [3]. In addition, segmenting a reliable vehicle logo candidate region from a complex background is also a critical factor for vehicle logo detection. 

Traditional machine learning methods design different handcrafted features to train a detector that is easy to understand and implement. Commonly used traditional features include feature points, edges, and invariant moments. However, detectors with specially designed features can only solve a single problem, which is inadequate to recognize vehicle logos under various complex environmental situations, such as night illumination, logo tilt, and bad weather. Deep learning-based object recognition methods have recently become dominant in computer vision tasks. However, only a few studies have contributed to VLR despite the wide use of convolutional neural networks (CNNs) for vehicle detection [4,5], vehicle model recognition [6,7], license plate recognition [8], Compression artifacts reduction [9], human action recognition [10], object tracking [11]. CNNs can learn different features from large-scale data and do not require human intervention during training. Hence, the outstanding performance of CNN in VLR tasks cannot be disregarded. Many researches [12,13,14] have improved their network by increasing the depth of the network or designing a good architecture. CNN-based methods have achieved immense success in many fields, but there is still much space for improvement in objects detection. The Faster R-CNN uses the top-most layer of a ConvNet (such as VGG, Resnet) to detect objects at different scales. However, few detail information of small objects are left on this layer after multiple levels of convolution. The SSD and YOLO v3 predict objects of different scales through different feature maps within a ConvNet. However, they all provide only one feature map for small object recognition, while other feature maps are more suitable for large object recognition. MTCNN [15] is a multi-task cascaded architecture with three deep convolutional networks, which use the inherent correlation between detection and key points to improve performance. However, some filters in convolution layers lack diversity that may result in some limit on the performance of multi-class objection detection and classification tasks. 

For the majority of the existing CNNs, numerous anchors are generated in the network for object detection and classification. However, the low match rate between small ground-truth logos and predicted anchors will result in small objects contributing less to feature learning during network training. To alleviate this problem, an enhanced matching method based on constrained region segmentation and copy-pasting strategy is proposed to improve match rate of small logos. Next, a SSFPD network is carefully designed in this paper to further improve the accuracy of small object recognition. In proposed SSFPD network, a reduced ResNeXt is used as base network to efficiently extract initial features. In addition, the Feature Pyramid Networks is built to provide more semantic information for the final SSFPD network, which increases recognition performance significantly. The major contributions of this research are as follows.

(1) An enhanced matching approach based on constrained region segmentation and copy-pasting strategy is proposed to improve the contribution of small objects to feature learning in network training, which is verified in the experiment.
(2) In order to further improve the detection accuracy, the proposed SSFPD network not only uses a better feature network to improve the capability of feature extraction, but also adds more semantic information about the small object for the prediction process.
(3) In this paper, a large common vehicle logo dataset (CVLD) containing various manufacturers is generated to evaluate the proposed method.

The remainder of this paper is organized as follows: Section 2 reviews the researches related to vehicle logo recognition. Section 3 presents the overview of the proposed method. Section 4 introduces constrained region extraction and enhanced matching for small objects. Section 5 provides a detailed description of the proposed SSFPD network. Section 6 presents the experiment results of the proposed method. Lastly, Section 7 presents the conclusions and future research.

## 2. Related Works

A typical vehicle logo recognition task often contains two processes: precise vehicle logo detection (VLD) and classification (VLC). VLD aims to find the locations of a logo in an image, VLC aims to assign a certain logo class label to each detected vehicle logo. This section discusses previous research on these two aspects.

### 2.1. Vehicle Logo Detection

The direct detection of vehicle logos is difficult because of the huge differences in the texture features, colors, and shapes among these logos. The most common method is extracting a candidate region containing vehicle logos through prior knowledge and using a coarse-to-fine method [16] thereafter to precisely locate these logos. Vehicle license plates and headlights are obvious objects in cars that help to define the size of the rectangular candidate region. The success of license plate location (LPL) technology [17,18] has prompted some research [19,20] to first detect the license plate and eventually use the topological relationship between the license plate and vehicle logo position to extract a candidate region that contains the logo. Thereafter, morphological methods [21,22,23] such as edge detection and color segmentation, are applied in the candidate region to accurately locate the logo. However, this method is unreliable because the distance between the license plate and logo is not fixed in different types of vehicles. The recognition rate will be immensely reduced if we extract a candidate region that does not contain the logo or only contain part of it. Moreover, this approach is not valid for vehicles that have no license plate. To alleviate this problem, Psyllos et al. [24] proposed an enhanced scale-invariant feature transform (SIFT)-based feature matching scheme. They first used the coordinates of the detected license plate to extract the vehicle mask (an area containing the headlights, the radiator grille, and the vehicle logo), which took about 100 ms. Next, a method based on phase consistency feature map (PCFM) calculation is performed to search near the middle of vehicle mask for vehicle logo location. The PCFM calculation process requires about 250 ms. Finally, a multiple feature matching (MFM) method took 1020 ms to recognize the vehicle logo. The MFM method can recognize the object by key point obtained from the observed part of the logo. In [25], a patch-based method for vehicle logo detection is proposed, which represent the coarse region of the logo with patch intensity and weight matrix. Hsieh et al. [26] applied a symmetrical descriptor to determine the region of interest of each vehicle from the road. However, these approaches rely heavily on a symmetrical front-view vehicle image. That is, these methods easily fail when the vehicle in the picture is tilted.

### 2.2. Vehicle Logo Classification

Traditional VLC methods mainly include feature point, template matching, edge feature, HOG feature, and support vector machine (SVM), among others. Template matching is the most basic and common matching method in image processing. Wang et al. [16] used the template matching method to classify different vehicle manufacturers. However, template matching is limited mainly because the template can only move in parallel. If the vehicle logo in the original image rotates or changes in size, then the algorithm is invalid. In [27], dense SIFT features are extracted to identify vehicle logos. Psyllos et al. [24] proposed an enhanced SIFT-based feature-matching scheme, which achieved better results than the standard SIFT-based feature matching method. These methods based on feature points can work well for a transformed image, but their drawback is that the resolution of the input image must be fixed. In addition, many systems use edge detection to deal with gray-level images. A shape-based method is proposed in [22] for vehicle logo classification. However, edge features are unreliable when the image resolution is low. Given that the HOG feature can maintain good invariance to geometric and optical deformations in images, the method [28] of combining HOG features with SVM classifiers has been widely used in vehicle logo recognition. Chen et al. [1] applied sparse representation for the application of vehicle manufacturer recognition. Cyganek et al. [29] presented a vehicle logo recognition system based on the novel combination of tensor-based feature extraction and an ensemble of tensor subspace classifiers. Peng et al. [19] proposed an approach based on statistical random sparse distribution (SRSD) feature and multiscale scanning to deal with poor-quality images captured from urban crossings. In general, traditional machine learning methods are laborious and require well-designed handcrafted features to accomplish the classification task.

CNN-based methods have achieved immense success in graphics and image processing tasks, which can be trained without any hand-designed features. In [20], a CNN-based system is proposed, which uses pretraining strategy to improve the performance of vehicle logo recognition. Although only a few researches have used the CNN-based method to recognize vehicle logos, the power of CNNs in small object detection and recognition cannot be disregarded. Li et al. [30] designed a perceptual generative adversarial network (perceptual GAN) that can effectively distinguish small and large targets in an image. A shifted single shot detector is proposed in [31] to address the misdetection caused by small instances located at gaps between default boxes. Furthermore, some methods [32,33,34] have provided additional information for small object recognition by cropping the corresponding context region surrounding the proposal region. Although many studies on small object detection and classification have been conducted, the accuracy of small object recognition and stability of small target recognition systems in complex environments can be further improved.

## 3. Overview of Proposed Method for Vehicle Logo Recognition

**Figure 1:**
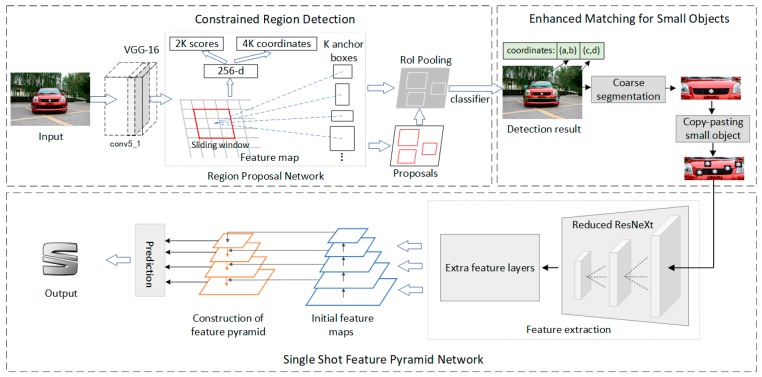
Framework of proposed method for vehicle logo recognition.

The overview diagram of our proposed VLR method is illustrated in Figure 1. The proposed method consists of three main parts. The first part is the constrained region detection module based on Faster-RCNN, which is used to extract the position information of the car head and tail. The entire image is forwarded through the VGG-16 network to obtain the last feature map (output of conv5/conv5_1 layer) with sufficient semantic information. Region proposal network is applied to the final feature map to generate numerous region proposals. Thereafter, the RoI pooling extracts the feature map of the target area through the generated region proposals. The accurate position information for the constrained region can be obtained through the classifier. The second part is enhanced matching for small objects, which consist of constrained region segmentation and copy-pasting strategy. By using the coordinates of the upper left and lower right corners of the detected candidate region, a constrained region containing the vehicle logos can be cropped from the original full image. Then, the copy-pasting strategy is adopted in the cropped image. The copy-pasting strategy is used in the training images to increase the overlap rate between the small ground-truth objects and predicted anchors in the CNN-based network. To make small objects sufficiently appear in each image that contains them, each image from the training set is augmented by copy-pasting vehicle logo many times. The enhanced matching method will be used on the SSFPD network proposed in third part. For the feature extraction module, a reduced ResNeXt network with high classification ability is adopted as base network to retain additional detailed information. A few additional feature layers are also added behind the ResNeXt to extract the initial feature. However, the generated initial feature maps with semantically weak features are insufficient for small object detection tasks. Therefore, a new feature pyramid with high-level semantics feature map is built on the network. These high-level semantic feature maps merged detailed information from the initial feature maps, which is beneficial for small objects recognition. The final result will be obtained through prediction on each high-level semantic feature map.

## 4. Logo Location Based on Constrained Region Detection and Enhanced Matching Method

### 4.1. Constrained Region Detection Using Faster R-CNN

Constrained region detection is critical to the vehicle logo detection. Traditional vehicle logo recognition methods first carefully detect the location of the logo and remove the background around the logo. The vehicle logo will then be recognized in the detected region. However, this method is highly dependent on a precise detection for logos location. If the logo is not accurately detected, for example, a part of the logo is not included in detected area, which will result in a low recognition rate. To alleviate this problem, this paper use Faster R-CNN to detect a large constrained region that containing the entire logo to increase the accuracy of logo detection for the subsequent recognition. To accurately extract the location information of the constrained region in various complicated situations, a suitable CNN-based object detection method should be chosen. The object detection methods can be divided into two groups: two-stage models (e.g., R-CNN [35], Fast R-CNN [36], and Faster R-CNN [37]) and one-stage models (e.g., YOLO [38] and SSD [39]). During network training, the one-stage model will generate lots of anchors for prediction, but only a few anchors are beneficial to the network learning. Most anchors that do not contain objects are unfavorable to network learning, which affects the overall accuracy. The two-stage model generates fewer anchors and the anchors that do not contain objects are less. Although anchors that do not contain objects can affect the overall accuracy, it is certainly not as serious as one-stage. Therefore, the accuracy of one-stage model is higher than one-stage model. As shown in Table 1, the Faster R-CNN achieves the highest accuracy. Therefore, the two-stage model is used to detect constrained region.

**Figure 2:**
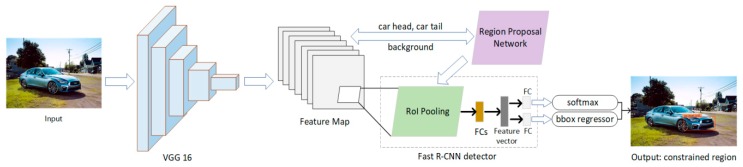
Our network of constrained region detection, include VGG16, region proposal network and Fast R-CNN detector.

The constrained region detection network consists of three modules (see Figure 2): Feature extraction module (VGG 16), region proposal network (RPN), and Fast R-CNN detector. Joint training is possible because RPN and Fast R-CNN detector can share the same convolutional layers. Therefore, a deep network (e. g., VGG 16) with 13 shareable convolutional layers is used in the Faster R-CNN for feature extraction. RPN is a fully convolutional network, which takes the last shared convolutional feature map of VGG 16 as input and generates a set of rectangular region proposals with a wide range of scales and aspect ratios. Candidate objects may or may not be included in these regional proposals. For the generation of region proposals, an n × n sliding window is convolved over the input feature map. Thereafter, two 1 × 1 filters were used for the task of box-regression (reg) and box-classification (cls). Moreover, 4k outputs that can encode the coordinates of the k bounding boxes are generated by the reg layer. The 2K classification scores output by the cls layer are used to estimate the probability whether each proposal contains a car head, car tail or the background. Fast R-CNN detector, which is the third module, is applied to refine the generated region proposals. The region of interest (RoIs) is taken as input in this module. The RoI pooling layer converts features in the region proposals into fixed-length feature vectors through max-pooling operation. Each RoI feature vector will be provided to a series of fully connected (FC) layers. The accurate location information of the constrained region can be obtained through the softmax and bounding-box regressor.

In general, the car head is an area that contains the headlights, radiator grille, and vehicle logo. The car tail is an area that contains the taillights and vehicle logo. In our experiment, 4000 images, including front-view images and rear-view images, are used to evaluate the effectiveness of the proposed method. The car head and car tail of each image in the dataset are carefully annotated. The dataset is divided into two parts: training and testing sets of 3200 and 800 images, respectively. Results of different methods are shown in Table 1. The Faster R-CNN achieves high accuracy of 98.3% in the car head and 96.2% in car tail, thereby demonstrating that the constrained region can be detected accurately enough. In addition, the detected constrained region is a region much larger than the logo. Although tiny deviation existed in the detection of the car head and car tail, the most detected large region can still ensure that the logo will be located inside the constrained region. Therefore, the vehicle logo recognition error due to the deviation of detection of the car head and car tail can be ignored.

### 4.2. Enhanced Matching for Small Objects

**Figure 3:**
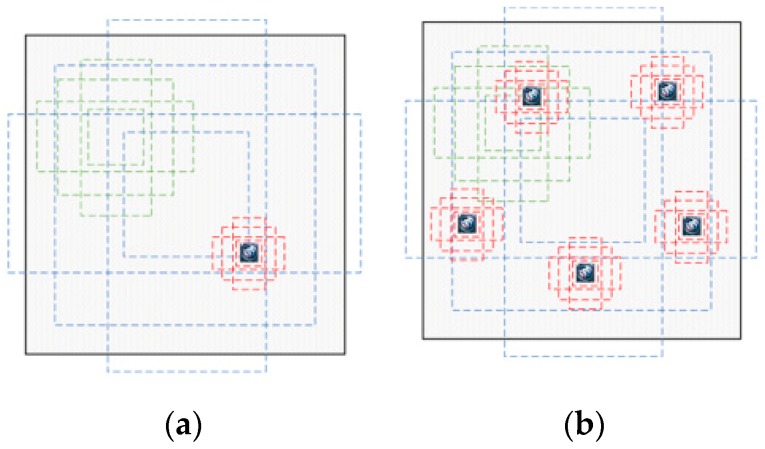
Anchors of different scales matching the ground truth objects; (**a**) Not using “copy-pasting” strategy; (**b**) Using “copy-pasting” strategy.

An enhanced matching method based on constrained region segment and copy-pasting strategy [40] is proposed in this section to improve the detection performance of small objects. Enhanced matching method is necessary in this paper. In most databases, only a small number of samples contain small objects, or the number of small targets is not enough in the sample containing small objects. These factors lead to the fact that the network has only a small part of the time to learn features of small objects during the training process. For majority of the existing CNNs, numerous anchors are generated by the anchor mechanism or region proposal network for object detection and classification. The greater number of anchors that positively match the ground-truth in the image, the greater the probability that the object will be detected. In other word, more positively matched anchors for small object can increase the contribution of small objects in calculating the loss function. To increase the number of positively matched anchors, this paper achieves data expansion by copying and pasting small objects into the training image multiple times. As shown in Figure 3a, there is less anchors positively matched with small logos. However, the number of positively matched anchors is significantly increased by using the copy-pasting strategy (see Figure 3b), which helps to improve the detection rate of small objects. 

**Figure 4:**
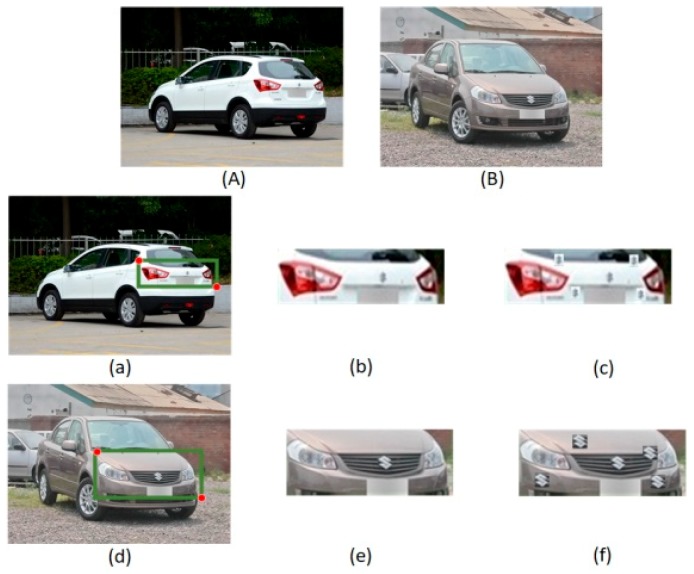
Process of constrained region segmentation and the copy-pasting strategy; (**A**) and (**B**) are original vehicle sample; (**a**) and (**d**) show the detected constrained region; (**b**) and (**e**) are the cropped constrained region; (**c**) and (**f**) show the images processed with “copy-pasting” strategy.

A cropping operation is performed to the extracted constrained region to generate a large rectangular area that includes the vehicle logos. Through constrained region segmentation, the search range and calculated consumption during vehicle location can be substantially reduced. For the copy-pasting strategy, the object labels provided in the dataset allow us to make a copy of any logo from its original location. Several aspects should be considered when pasting a copy of the vehicle logo. First, a pasted vehicle logo will never overlap with any other logos in the cropped image. Second, all small logos in each image are copied and pasted four times at random locations in the cropped image. Third, the copy-pasting strategy is only adopted in the training set, but the testing set remains unchanged. By using the enhanced matching method, the number of matched default boxes on small objects can be considerably increased, thereby increasing the contribution of small objects in calculating the loss function. Process of constrained region segmentation and copy-pasting strategy are shown in Figure 4. Rather than merely focusing on the front-view vehicle images as the majority of the existing researches do, the proposed VLR can be applied to the front-view and rear-view vehicle images. Vehicle samples captured from different angles are shown in Figure 4A,B. The constrained regions (see Figure 4a,d) detected by the Faster R-CNN are larger than the vehicle logo. By using the coordinates of the upper left and lower right corners of the detected candidate region, the constrained region containing the vehicle logos can be cropped (see Figure 4b,e). The training images augmented by the copy-pasting strategy are presented in Figure 4c,f. The transformation of annotation relative to an image window is a standard operation and can be done automatically by the corresponding code.

## 5. Proposed SSFPD Network

**Figure 5:**
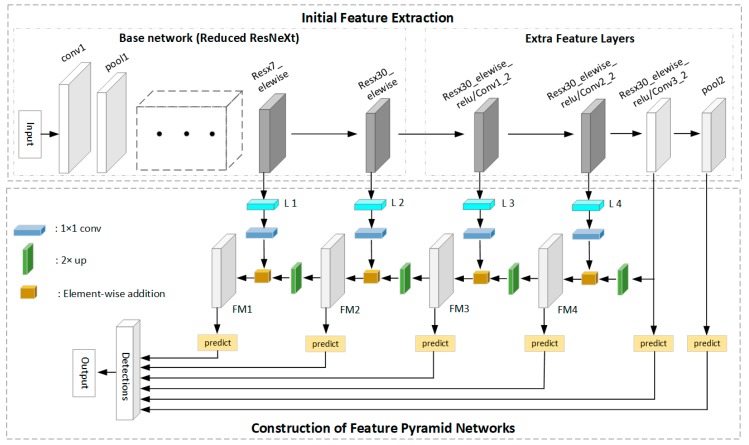
The framework of proposed SSFPD network.

The two-stage model first generates regional proposals, then classifies the candidate regions and refines the locations. However, the production of the region proposals is time consuming. The one-stage model has great advantages in speed, which does not require the region proposal stage and can directly generate the class probability and location of the object through a stage. Therefore, a novel one-stage framework is proposed for small logo recognition, which consists of two interconnected modules, initial feature extraction module and FPN network. The framework of the proposed SSFPD network is illustrated in Figure 5. 

### 5.1. Initial Feature Extraction 

**Figure 6:**
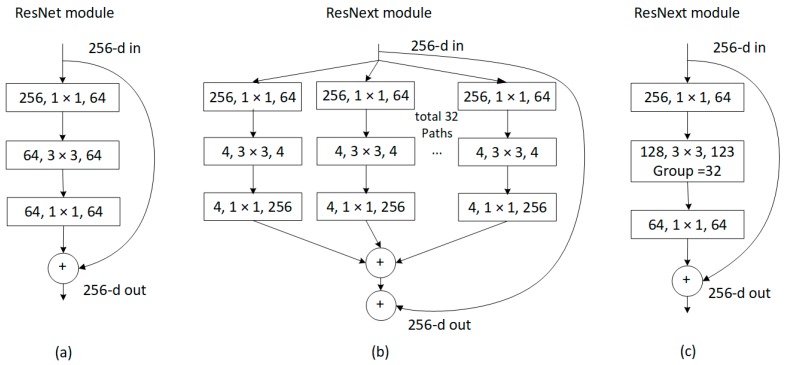
Different building blocks. (**a**): A block of ResNet (**b**): A block of ResNeXt with cardinality of 32. (**c**) A block equivalent to (b).

In the initial feature extraction module, the ResNeXt-101 is used as base network to generate the initial feature maps. ResNeXt, which was proposed by Xie et al. [41], adopts the optimization benefits conferred by the skip-connection of ResNet [10] and split-transform-merge strategy from the inception module [42]. A building block of ResNext is shown in Figure 6b. ResNeXt uses multi-branch architectures to replace the two-branch network of ResNet (see Figure 6a), thereby showing a strong feature extraction power. The transformations to be aggregated in the building block of ResNeXt have the same topology. Given that all paths share the same topology, the number of paths is isolated to produce a new dimension called cardinality. Different from other network with only two dimensions of depth and width, ResNeXt can gain accuracy through a considerably effective method of increasing cardinality. In our experiment, the cardinality is set to 32. Moreover, a equivalent building block with grouped convolutions (see Figure 6c) are used to make the module substantially succinct. ResNeXt-101 shows better accuracy than ResNet-101/152 [13], ResNet-200 [43], and Inception-ResNet-v2 [42] on the ILSVRC 2016 classification and detection tasks. Therefore, this successful model is a good choice for our base network.

Inspired by the design strategy of SSD [39], extra feature layers are provided to the end of the reduced ResNeXt. Different from the original SSD, the feature maps generated by the base network and added layers are used to construct high-level semantic feature layers in the FPN network instead of directly for the predicting detection. The architecture of ResNeXt-101 is shown in Table 2. Instead of using the full network and obtaining the feature map at the last layer (resx33_elewise layer), the output of the resx7_elewise layer and res30_elewise layer are taken as initial feature maps. If the res33_elewise layer is used, then a high risk of losing the small objects is present owing to the substantially small resolution of this layer. These two feature layers that we adopted have output shapes of [65 × 65] and [33 × 33], which are lager than an ordinary feature map (with 17 × 17 resolution) from the resx33_elewise layer in the original full network. Therefore, the outputs of the resx7_elewise and resx30_elewise layers are selected and extra feature layers are provided after the resx30_elewise layer. Furthermore, the extra feature layers decrease in size progressively and allow the feature extraction of objects at multiple scales. The four layers of Resx30_elewise_relu/Conv1_2, Resx30_elewise_relu/Conv2_2, Resx30_elewise_relu/Conv3_2, and pool2 are also used for the generation of the initial feature maps. Lastly, a feature pyramid network is generated on the basis of the initial feature maps to obtain additional semantic information. 

### 5.2. Generation of the FPN Network and Object Recognition 

To improve the accuracy of vehicle logo recognition, an FPN [44] network that was shown to be helpful for small object detection is adopted in our network. In our proposed SSFPD network, the building of an additional feature pyramid network is used to strengthen the features. For our proposed FPN, the detailed information in the shallow layers can be retained through the fusion of different levels of features on the carefully selected initial feature map. These initial features with large resolution and strong feature extraction capabilities will help improve the efficiency of the upsampling operation. In addition, in order to optimize memory consumption, the last two feature maps with sufficient semantic features in the initial feature maps will be used directly for prediction. 

**Figure 7:**
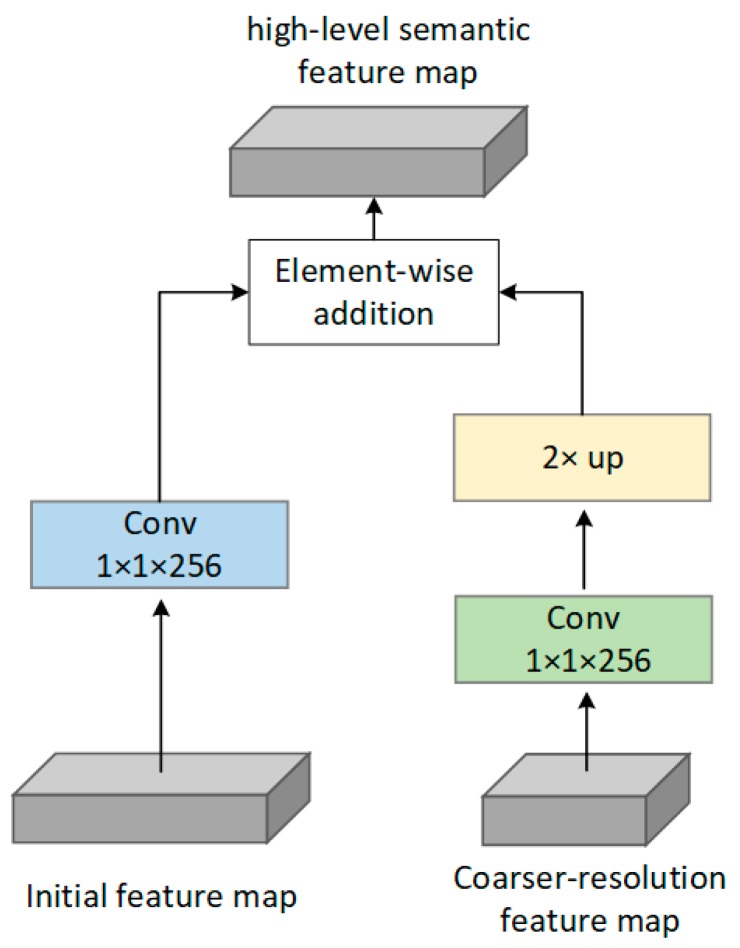
A building block of FPN.

The proposed FPN network is shown in Figure 5. Bottom–up pathway, top–down pathway, and lateral connections are used. A building block of FPN is shown in Figure 7. The bottom–up pathway is a feed-forward process on the base network and extra feature layers. Four initial feature maps output by layers of resx7_elewise, resx30_elewise, Resx30_elewise_relu/Conv1_2, and Resx30_elewise_relu/Conv2_2 are denoted as {L 1, L 2, L 3, L 4}, respectively, all of which will be enriched to create our pyramid. A 1 × 1 convolutional layer is used to reduce the channel dimension of the four initial feature maps to 256. The other two initial feature map output by Resx30_elewise_relu/Conv3_2 and pool2 are excluded in the pyramid because of their large memory consumption. The top–down pathway is accomplished by upsampling operation. By performing an upsampling with a factor of 2 on the coarser-resolution feature map (e.g., layer of Resx30_elewise_relu/Conv3_2, FM1, FM2, FM3, and FM4), the upsampled feature map will have the same size as the feature map generated by the next layer. Another 1 × 1 convolutional layer before the upsampling operation is used to ensure that the channel dimensions of the upsampled feature map is the same as the corresponding bottom–up map. Lastly, lateral connection is performed to merge the feature maps of the same spatial size from the bottom–up and top–down pathways. Therefore, a new pyramid consisting entirely of high-level semantic feature maps is built.

The tasks of detection predictions and object classification are performed on the multi-scale high-level semantic feature maps (FM1, FM2, FM3 and FM4) and two feature layers of Resx30_elewise_relu/Conv3_2 and pool2. With a set of convolution filters, each feature map used for prediction produces a fixed set of default boxes. To solve the problem of multiple-scale object detection, different default boxes are used with particular designed scales and five aspect ratios $\left(a_{r}=\left[1,\;2,\;3,1/2,1/3\right]\right)$. Six feature maps are adopted in the proposed network for prediction. The scale for each feature map is defined as follows:

(1) $$s_{k}=s_{\min}+\frac{s_{\max}-s_{\min}}{m-1}\left(k-1\right),\;k\in \left(1,6\right)$$

where $S_{\min}$ indicate that the scale in the lowest feature map is 0.2 and $S_{\max}$ represent that the scale in the highest feature map is 0.9. All feature maps in between are regularly spaced. In addition, the width $\left(w_{k}^{a}=s_{k}\sqrt{a_{r}}\right)$ and height $\left(h_{a}^{r}=s_{k}/\sqrt{a_{r}}\right)$ can be computed for each default box. 

During the training process, 13 labels are assigned to each groundtruth box according to such categories as vehicle logos and background. The overall objective loss function is defined as follows:

(2) $$L\left(x,\;c\;,l,\;g\right)=\frac{1}{N}\left(L_{\mathrm{conf}}\left(x,c\right)+{\alpha L}_{\mathrm{loc}}\left(x,l,g\right)\right)$$

where N is the number of matched default boxes, the confidence loss $L_{\mathrm{conf}}\left(x,c\right)$ is a softmax loss over 13 classes, and the localization loss $L_{\mathrm{loc}}\left(x,l,g\right)$ is the default bounding box regression for positive boxes.

## 6. Experiments

This section evaluates the proposed VLR method using self-collected and public vehicle logo datasets. A brief description of the dataset is first provided. Thereafter, the implementation details of the proposed SSFPD are introduced, along with some comparison experiments. Lastly, the result of the proposed VLR method and results obtained by our competitors are reported.

### 6.1. Date Set Descriptions

**Figure 8:**
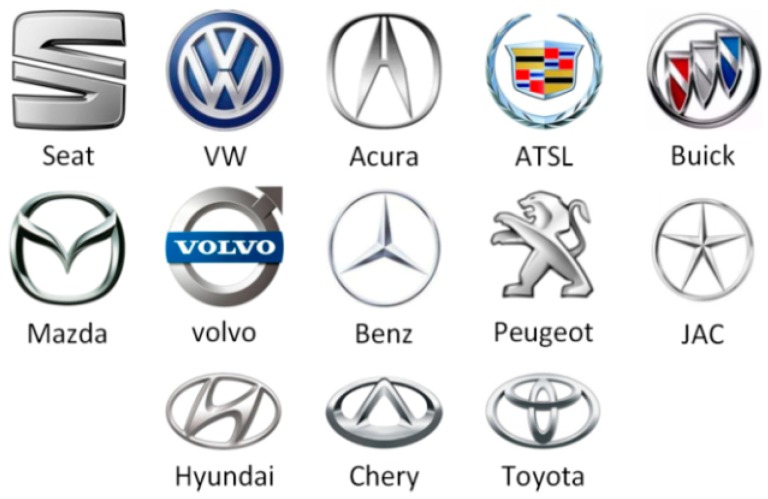
Samples of 13 logo classes.

A few public benchmarks are available for VLD and VLC. Psyllos et al. [24] presented a dataset of 1200 frontal area images segmented from the Medialab LPR Database. Peng et al. [18] created a dataset with 3370 images covering 56 logo categories. All images in this dataset are captured from cameras placed at some major intersections. Huang et al. [20] provided a dataset containing 11,500 logo images belonging to 10 manufacturers. However, these datasets are not widely used. The datasets provided in [19,24] are extremely small. Evidently, testing on a set with limited number of images is inadequate to prove the robustness of the VLR methods. The dataset provided by Huang et al. [20] is sufficiently large, but images in various complex natural environments are not included. Consequently, the preceding datasets are not suitable for evaluating practical VLR methods. This paper generates a self-collected dataset called Common Vehicle Logo Dataset (CVLD). The images in the CVLD dataset are carefully selected from two vehicle-related benchmarks: Chinese City Parking Dataset (CCPD) [45] and Comprehensive Cars dataset (CompCars) [46]. The CVLD dataset has a total of 14950 images, including various poor outdoor imaging situations. The 13 classes of vehicle logos in this data set are shown in Figure 8. The resolution of these logos ranges from 10 × 10 pixels to 150 × 150 pixels, the majority of which are lower than 50 × 50 pixels.

**Figure 9:**
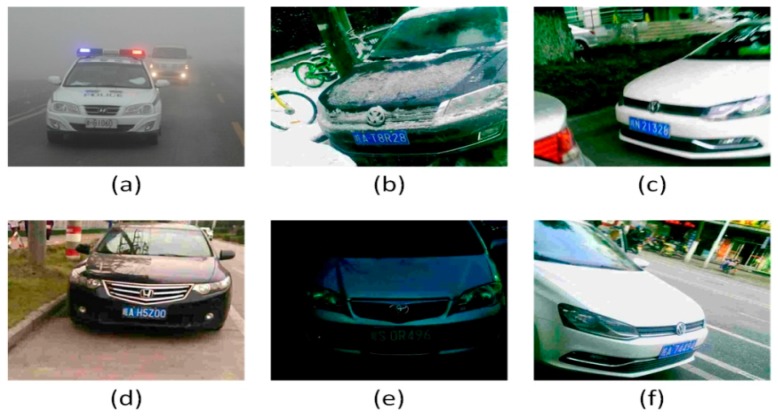
Some example in CVLD dataset: (**a**) fog (**b**) snow (**c**) rain (**d**) day (**e**) night (**f**) tilt.

In our experiment, 13,000 images were used for training and 1950 images were used for testing. That is, each manufacturer has 1,000 images as training samples and 150 images as testing samples. Moreover, three additional testing sets with various situations are used to further demonstrate that the proposed method is considerably appropriate for real-world application. Table 3 shows that the CVLD_weather test set contains 920 images in the bad weather of fog, snow, and rain. The CVLD_night test set has 665 images at night. The CVLD_tilt test set includes 750 images with horizontal and vertical tilts of 15° to 45° and 15° to 45°, respectively. Some example images in our dataset are shown in Figure 9.

### 6.2. Implementation Details and Model Analysis

The final VLR method is written in Python with OpenCV and Caffe library. OpenCV is used to implement constraint region segmentation, background removal, and copy-pasting operations. The proposed SSFPD network is well trained with the support of Caffe. Our recognition system runs on a PC with a 4-core 3.3 GHz CPU and Nvidia Quadro P5000 GPU. An enhanced matching method is adopted in the proposed SSFPD network. The original training images are processed with constrained region segmentation and copy-pasting strategy before they are inputted into the network. For the network training, the input image resolution is set to 513 × 513. The base network ResNeXt is pretrained on the ILSVRC CLS-LOC dataset [47]. A series of high-level semantic feature map generated by the FPN network are used to bring additional context information for the prediction stage. Upsampling of resx30_elewise_rule/con3_2, FM 4, FM 3, and FM 2 is done by stride-2 convolutions in the 3×3 layer. Table 4 shows the resolution of each feature maps used for prediction. Although both SSFPD and YOLOv3 used multi-scale fusion methods, they differ in object prediction. The YOLOv3 fuse feature maps of three different scales (52×52, 26×26, 13×13) for object prediction, but only one feature map with large scale of 52×52 is suitable for small object prediction. For proposed SSFPD network, five different scales (65×65, 33×33, 13×13, 9×9, 5×5) are fused to add more semantic information for object prediction, and two feature maps with scale of 65×65, 33×33 are provided to small object recognition. Compare to YOLOv3, the proposed SSFPD not only fuse more feature maps to pass more semantic information for the final network, but also provides more large-scale feature maps to improve the accuracy of small object detection. To fine-tune the resulting model, we use SGD with a basic learning rate of 0.001 and a momentum of 0.9. The batch size is set to 1. After every 50,000 of iterations, the learning rate is reduced by a factor of 10. The training process stopped after 200,000 iterations.

To understand SSFPD better, control experiments are carried out to examine how each component affects the final performance. The parameter settings and input size (513 × 513) are consistent across all experiments, except for specified changes to components. Table 5 shows the experiment results based on various design choices and components. The final used architect of SSFPD achieved the best accuracy of 93.79%, which contains the components of resx7_elewise layer, resx30_elewise layer, FPN and copy-pasting strategy. Then, the effectiveness of each component is verified. As described in Section 4.2, the copy-pasting strategy improves the accuracy by increase the match rate between small ground-truth objects and the predicted anchors during network training. If the copy-pasting strategy is not adopted, the performance drops by 2.1% (91.69% vs. 93.79%). As described in Section 5.1, the resx7_elewise layer and the resx30_elewise layer are selected as initial feature map to retain more detail information about small objects. If they are replaced by resx33_elewise layer, the result drops by 3.83% (89.96% vs. 93.79%). As described in Section 5.2, using a FPN can provide detail information of small logos for network. If the initial feature maps in ResNeXt are directly used for object prediction, the performance for small logo recognition will be decrease greatly (86.47% vs. 93.79%). 

### 6.3. Experimental Results

#### 6.3.1. Comparison of Different Methods 

The proposed method was compared with several different CNN-based networks: Faster R-CNN [37], YOLO v3 [38], SSD [39], Pretraining CNN [20] and MTCNN [15]. Note that all comparison networks in this experiment used the same CVLD dataset. In our experiment, some adjustments are made to the competitive network for small object detection task. For SSD, Faster R-CNN and YOLO v3, the original backbone architecture is changed to the reduced ResNeXt-101 and the higher resolution maps are selected for object prediction. The adjusted YOLO v3 can predict small logos on three feature maps with scales of 65×65, 33×33 and 13×13. The last feature layer for detection in the adjusted faster r-cnn has a higher resolution of 17×17. The resolution of the feature map for small objects recognition in SSD is adjusted to 65×65 to retain more detail information. In addition, the MTCNN is trained to predict front/rear of the vehicle and the corners location of the vehicle logo. The results are shown in Table 6. With 513×513 input, the proposed SSFPD network requires about 52 ms (20 fps) to processes an image. The memory consumption of network’s weight is 169.1 M. The average time-consumption of our proposed method is just a little bit bigger than other methods, but our proposed SSFPD achieved the best accuracy.

The proposed method and SSFPD network are also validated on another public dataset provided by Huang et al. [20]. Table 7 shows that the proposed methods are significantly better than the methods of MFM [24] and M-SIFT [47] in term of mAP and testing time. Compared to the pre-training CNN [20], the testing time of proposed method is slower. However, the proposed SSFPD with enhanced matching method is better than the pre-training CNN with a higher mAP.

#### 6.3.2. Performance on Various Complex Conditions

**Figure 10:**
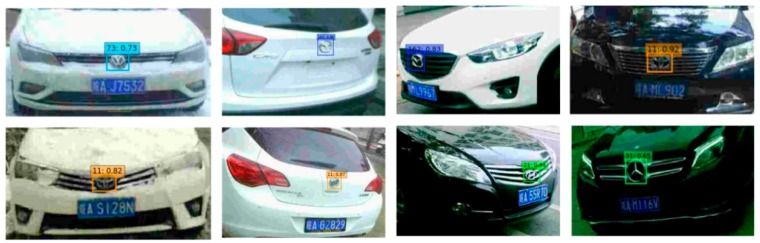
Success examples under complicated environments.

Recognizing small logos in complex conditions is still a challenge. Some methods are able to achieve acceptable results under good lighting conditions, but they do not perform well in detecting small logos under various more complex conditions. To further evaluate the robustness of the proposed method, different methods were tested on three testing sets with different environmental conditions. As shown in Table 8, the proposed method achieves better results than others in small logos detection. In addition, Table 9 lists the recall, precision, and accuracy of the proposed method on different test data sets. True positive (TP) means the positive samples are recognized as vehicle logos. True negative (TN) indicates that the positive samples are not recognized as vehicle logos. False positive (FP) means that the negative samples are recognized as vehicle logos. False negative (FN) indicates that the negative samples are not recognized as vehicle logos. Parameter recall, precision, and accuracy are used to evaluate the performance of the proposed method of the SSFPD network with enhanced matching method and calculated as follows:$\;\mathrm{Recall}=\frac{\mathrm{TP}}{\mathrm{TP}+\mathrm{FN}}$, $\mathrm{Precission}=\frac{\mathrm{TP}}{\mathrm{TP}+\mathrm{FP}}$ and $\mathrm{Accuracy}=\;\frac{\mathrm{TP}+\mathrm{TN}}{\mathrm{TP}+\mathrm{TN}+\mathrm{FP}+\mathrm{FN}}$. The results show that the accuracy of the three testing sets remains above 80% and error recognition and missing recognition rate are low. The results prove that the proposed method is suitable for real-world applications. Some results of the proposed method under the complicated environments are shown in Figure 10. 

## 7. Conclusions

This paper proposes a VLR method based on enhanced matching for small objects, constrained region, and SSFPD network. Faster R-CNN is used to extract a constrained region containing vehicle logos. An enhanced matching approach based on constrained region segmentation and copy-pasting strategy is proposed to improve the contribution of small objects to feature learning in network training. In order to further improve the recognition accuracy, the proposed SSFPD network not only uses a better feature network to improve the capability of feature extraction, but also adds more semantic information about the small object for the prediction process. The reduced ResNeXt is used to improve the classification performance of the network and retain additional detailed information for small vehicle logo detection. In addition, the feature pyramid networks module is adopted to bring in more semantic context information to build several high-level semantic feature maps, which effectively improves recognition performance. Extensive experiments demonstrated that the proposed method performs better than the existing methods in the recognition of small vehicle logo and more appropriate for application in complex environments. In future researches, vehicle logos and vehicle model will be considered together to identify a vehicle. Real-time vehicle logo detection by optimizing the network structure will be investigated as well.

## Figures and Tables

**Table 1:** Results of different methods for car head and car tail detection.

Methods	Car Head	Car Tail
SSD [39]	95.4%	92.7%
YOLO [38]	96.1%	93.5%
Faster R-CNN [37]	98.3%	96.2%

**Table 2:** The architecture of ResNeXt-101.

Layers	Output
pool	129 × 129
resx1_eleswise to resx7_eleswise	65 × 65
resx8_eleswise to resx30_eleswise	33 × 33
resx30_eleswise to resx33_eleswise	17 × 17

**Table 3:** Test images sets in various complex environments.

Test Set	Conditions	Image Amount
CVLD_weather	Fog, Snow and Rain	920
CVLD_night	Night	665
CVLD_tilt	Tilt	750

**Table 4:** The resolution of each feature maps used for prediction.

Layer	Resolution
FM 1	65×65
FM 2	33×33
FM 3	13×13
FM 4	9×9
Resx30_elewise_relu/Conv3_2	5×5
Pool 2	1×1

**Table 5:** Performance comparison of different design for SSFPD.

Architect of SSFPD	The Final Used Architect	Not Using Copy-Pasting Strategy	Using Resx33_elewise, Not Using Resx7_elewise and Resx30_elewise	Not Using FPN
mAP	93.79%	91.69%	89.96%	86.47%

**Table 6:** Accuracy comparison on CVLD dataset.

Methods	Network	mAP	Testing Time	Memory	Input Resolution
SSD [39]	VGG 16	79.2%	23 ms	110.7 M	300×300
	ResNext-101	85.7%	45 ms	133.1 M	513×513
Faster R-CNN [37]	VGG 16	81.9%	30 ms	217.9 M	300×300
	ResNext-101	86.3%	56 ms	346.3 M	513×513
YOLO v3 [38]	DarkNet-53	82.7%	20 ms	226.6 M	300×300
	Resnext-101	89.8%	49 ms	346.3 M	513×513
Pre-training CNN [20]	---	88.9%	21 ms	88.6 M	513×513
MTCNN [15]	---	90.4%	34 ms	101.5 M	513×513
Proposed method	ReaNext-101	91.7%	52 ms	169.1 M	513×513

**Table 7:** Accuracy comparison on public dataset.

Methods	mAP	Testing Time
MFM [24]	94%	1020 ms
M-SIFT [48]	94.6%	816 ms
MTCNN [15]	98.76%	35 ms
Pre-training CNN [20]	99.07%	12 ms
proposed method (SSFPD)	99.26%	52 ms
proposed method (SSFPD + enhanced matching)	99.52%	108 ms

**Table 8:** Accuracy comparison on complex conditions.

Testing Set	Accuracy		
	M-SIFT [48]	Pretraining CNN [20]	Proposed Method
CVLD_weather	74.8%	77.4%	80.6%
CVLD_night	77.6%	82.1%	84.0%
CVLD_tilt	79.9%	83.7%	86.5%

**Table 9:** Performance on different testing sets.

Testing Set	Real Result	Prediction		Recall	Precision	Accuracy
		Positive	Negative			
CVLD_weather	True	764	18	83.04%	95.98%	80.61%
	False	32	156			
CVLD_night	True	579	12	87.06%	95.70%	84.06%
	False	26	86			
CVLD_tilt	True	663	15	88.4%	97.36%	86.59%
	False	18	87			
